# Use of artificial intelligence for detecting left ventricular dysfunction and predicting incident heart failure risk

**DOI:** 10.1002/ehf2.15442

**Published:** 2025-10-15

**Authors:** Anna Węgrzyn‐Witek, Monika Przewlocka‐Kosmala, Wojciech Kosmala, Thomas H. Marwick

**Affiliations:** ^1^ Jan Mikulicz Radecki University Hospital Wroclaw Poland; ^2^ Wroclaw Medical University Institute of Heart Diseases Wroclaw Poland; ^3^ Baker Heart and Diabetes Research Institute Melbourne Victoria Australia; ^4^ Menzies Institute for Medical Research Hobart Tasmania Australia

**Keywords:** artificial intelligence, echocardiography, electrocardiography, heart failure, prevention

## Abstract

Effective medications are available for the prevention of heart failure (HF). While their use is indicated in patients with risk factors, engagement and adherence among ‘at risk’ individuals is challenging, as it is with atherosclerotic heart disease prevention. The detection of patients with subclinical cardiac dysfunction could provide a subgroup at heightened risk, warranting more intensive disease management programmes. The process of screening the aging population is a huge task that could be facilitated using artificial intelligence (AI) to identify clinical risk, select ‘at risk’ individuals by using AI to enhance the value of electocardiography, and facilitate the non‐expert acquisition and interpretation of echocardiography. This review, informed by a search of the recent literature, explored how such an AI‐informed pathway could permit HF screening to occur in the community—maximizing access and minimizing cost.

## Background

Heart failure (HF) is a major public health concern, affecting over 64 million individuals worldwide. In developed countries, its prevalence among the general adult population is estimated at 1–2%, with rates rising markedly with advancing age.^1^ The survival rate in chronic HF progressively declines over time, dropping to 87%, 73%, 57% and 35% at 1, 2, 5 and 10 years, respectively.^2^ A meta‐analysis of echocardiographic studies revealed that the prevalence of left ventricular (LV) dysfunction in the general population exceeds that of overt HF, reaching 4.2% overall and up to 11.8% among individuals aged 65 years and older.^3^ This difference is attributed to subclinical (or ‘stage B’) HF—defined by LV dysfunction and structural remodelling—with 76% of affected individuals exhibiting preserved ejection fraction (HFpEF).^4^

**Figure 1:**
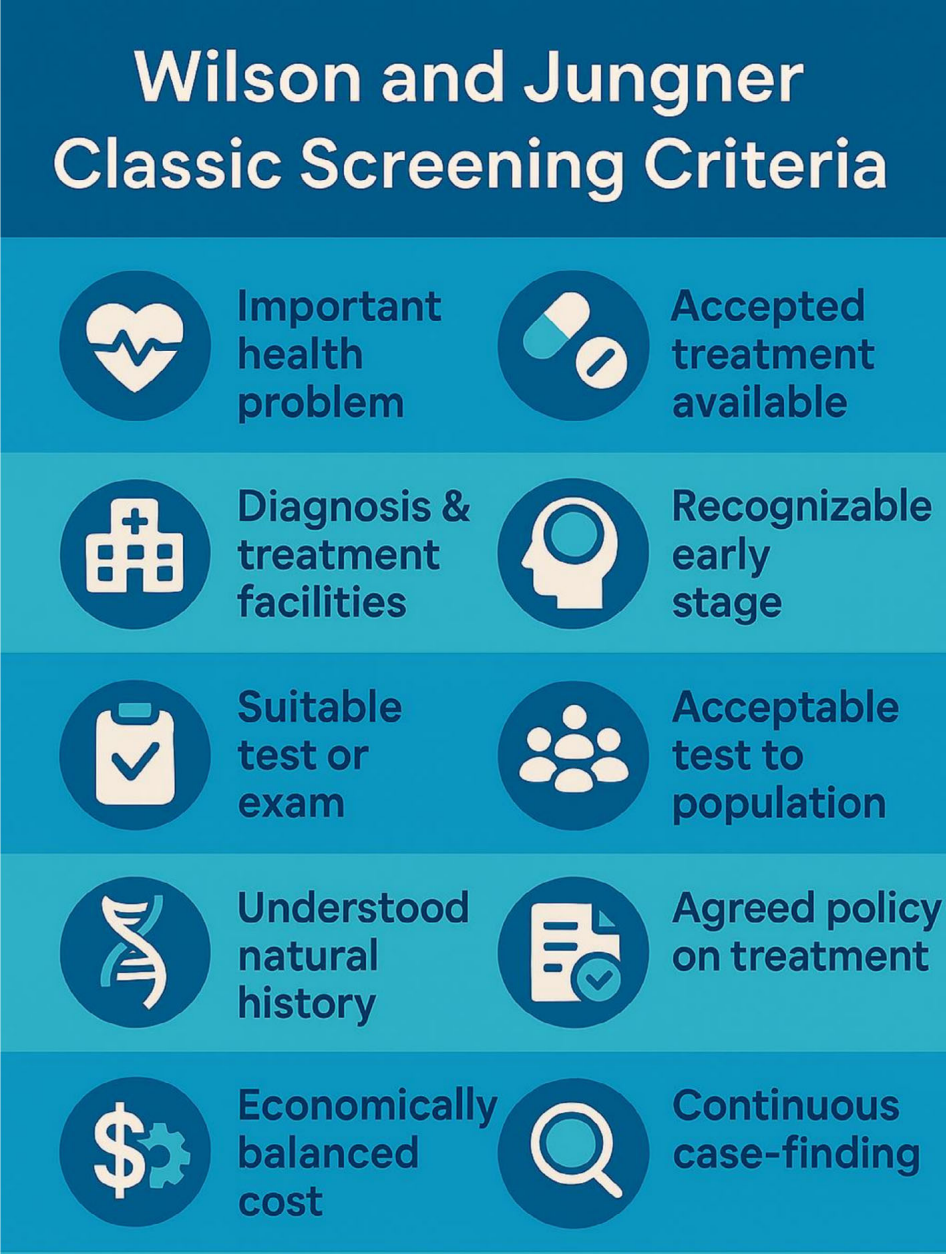
Wilson and Jungner classic screening criteria.

Early recognition of asymptomatic stages of HF allows for risk assessment and targeting the implementation of cardioprotective strategies to individuals in whom lifestyle changes and medications may delay or prevent the development of overt HF.^5^ Effective early treatment can reduce the frequency of hospitalisations, which are prevalent in advanced stages of heart HF and represent a significant economic burden on healthcare systems worldwide. Recent data from the STAAB trial, showing an even higher than previously reported prevalence of SBHF at 17% in the urban population studied, further underscores the urgent need to screen for individuals from this category.^6^ Interestingly, about 1/3 of patients with SBHF lack CV risk factors.^7^ On these grounds, it seems that the evaluation of risk in those with risk factors may be insufficient, and a broader screening strategy should be considered. Such an approach would satisfy the requirements for screening enumerated by Wilson and Jungner: an important condition with an understandable natural history, a recognizable latent stage, with a suitable test accepted, and early treatment that is acceptable to the population (*Figure* *1*).^8^ The conventional approach to the identification of SBHF would be to use a clinical risk score to select patients for additional testing with natriuretic peptides or echocardiography.^9^

In this narrative review, we sought the possible contribution of artificial intelligence to such a process. We performed a targeted literature search between November 2024 and March 2025. The search strategy included the following keywords: (‘Heart Failure’ OR ‘HF’) AND (‘Risk Assessment’ OR ‘Risk Prediction’ OR ‘Prognosis’) AND (‘Artificial Intelligence’ OR ‘AI’ OR ‘Machine Learning’ OR ‘ML’ OR ‘Deep Learning’). A total of 997 records were retrieved. After screening for clinical relevance and methodological quality, 111 articles were selected for a full‐text evaluation. Of these, 44 studies were ultimately included in the final synthesis. The reviewed literature focused on the application of artificial intelligence in the clinical assessment and diagnosis of HF, encompassing domains such as symptom evaluation, laboratory testing, electrocardiography (ECG) and echocardiography.

## Artificial intelligence methods

Artificial intelligence (AI) provides the capability to perform human intelligence‐dependent tasks such as pattern recognition and identification, planning, understanding language, recognizing objects and sounds, and problem‐solving using tools such as machine learning (ML), and its subtype deep learning (DL). The essential requirements for the creation of ML tools are high quality data and ML computational techniques, tailored to data analysis. Potential data sources include images, electronic health records (EHR), prescribing, outcomes including mortality, information about patient habits (e.g., diet, lifestyle, activity—derived from wearable devices), and big data from biobanks, bioresources and genomics.^10^ These highly‐dimensional datasets may allow the capture of unrecognized variables and interactions associated with HF. Supervised learning (*Table* *A1*) divides the entire process into smaller, repeatable cycles. Each iteration consists of planning, analysis, design, implementation and testing stages to discern patterns of data that fit a particular outcome of interest. It uses a pre‐labelled database to predict the outcomes of future events. Unsupervised learning (*Table* *A2*) uses unclassified databases to predict specific relationships within a set of data. The programme does not attempt to match the data to the result, but instead simply tries to identify potential consistent patterns in the data. This approach gives AI the ability to learn rules and identify patterns progressively from large datasets, without being explicitly programmed or any a priori assumptions.

While classical models (random survival forest^11^ and extreme gradient boosting [XGBoost]) can demonstrate high performance in various prediction tasks, modern deep learning architectures [convolutional neural network (CNN), residual network (ResNet) and transformer] offer additional advantages, such as better utilization of sequential data, integration of multimodal information sources, and greater flexibility in adapting to new data. Nevertheless, their complexity may pose a barrier to clinical implementation.

## Clinical detection of HF risk and risk factors

The most common risk factors for developing heart failure are increased blood pressure, ischaemic heart disease, diabetes, obesity, smoking, hypercholesterolaemia and lifestyle factors (reduced physical activity, unhealthy diet and stress). A variety of risk scores—analogous, but less widely used than risk scores for atherosclerotic CV disease—have been developed.^12^ The older scores, including FHS (Framingham Heart Study),^13^ ARIC (Atherosclerosis Risk in Communities)^4^ and Health ABC HF risk scores^14^ were derived from single cohorts and therefore have limited generalizability. Much larger datasets have been used to develop the more recent PCP‐HF (Pooled Cohort Equation‐HF)^15^ and PREVENT‐HF (Predicting Risk of Cardiovascular Disease Events–Heart Failure)^5^ scores, which have been validated to predict the risk of developing HF and categorize patients into low, intermediate and high risk. *Table* *1* summarizes the discrimination with these tools, with most AUCs in the range of 0.7–0.8, implying acceptable—but not excellent—discrimination.

**Table 1:** Prediction of HF with standard clinical HF scores

Study cohort	Age/clinical	Race	Follow‐up	Internal validation	External validation
PREVENT‐HF^5^	Age 30–79 CVD‐Free	White (W/M): 78/80% Black: 10/8% Hispanic: 6/5,3% Asian: 2,6/2,5%	5 years	Total CVD C‐statistic: Women: 0.789 (0.778–0.810) Men: 0.745 (0.734–0.760) ASCVD C‐statistic: Women: 0.774 (0.743–0.788) Men: 0.736 (0.710–0.755) HF C‐statistic: Women: 0.830 (0.816–0.850) Men: 0.809 (0.777–0.827)	Total CVD C‐statistic: Women: 0.794 (0.763–0.809) Men: 0.757 (0.727–0.778) ASCVD C‐statistic: Women: 0.774 (0.743–0.788) Men: 0.736 (0.710–0.755) HF C‐statistic: Women: 0.830 (0.816–0.850) Men: 0.809 (0.777–0.827)
PCP‐HF^15^	Age 30–79 CVD‐Free	White: 78%	12 years	White men AUC: 0.79 White women AUC: 0.85 Black men AUC: 0.71 Black women AUC: 0.78	White men AUC: 0.80 (PREVEND) White women AUC: 0.87 Black men AUC: 0.74 (JHS) Black women AUC: 0.76
MESA^16^	Age 45–84 CVD‐Free	White‐ 39%, Black‐ 28%, Hispanic‐ 22%, Chinese‐ 12%	5 years	AUC: 0.87	‐
FHS^13^	Age 45–94 Prior CVD	White‐ 100%	38 years	‐	AUC: 0.614 (ARIC) AUC: 0.735 (men), AUC 0.684 (women); Health ABC
ARIC^4^	Age 45–64 Prior CVD	White‐ 73%	15 years	AUC: 0.797	‐‐
Health ABC^17^	Age 70–79 Prior CVD	White‐ 59%	6 years	AUC: 0.72	AUC: 0.785 (ARIC) AUC: 0.74 (CHS)
International Collaboration of HF subtypes (FHS, CHS, PREVEND)^18^	Age 30–79 Prior CVD	White: 95%	13 years	HFpEF: AUC: 0.79 HFrEF: AUC: 0.80	HFpEF: AUC: 0.76; HFrEF: AUC: 0.76 (MESA)

ARIC – Atherosclerosis Risk in Communities; ASCVD ‐ Atherosclerotic Cardiovascular Disease; AUC – Area Under the Curve; CHS – Cardiovascular Health Study; CVD – Cardiovascular Disease; FHS – Framingham Heart Study; Health ABC – Health, Aging, and Body Composition Study; HFpEF – Heart Failure with Preserved Ejection Fraction; HFrEF – Heart Failure with Reduced Ejection Fraction; JHS – Jackson Heart Study; MESA – Multi‐Ethnic Study of Atherosclerosis; PCP‐HF – Pooled Cohort Equations to Prevent Heart Failure; PREVEND – Prevention of Renal and Vascular End‐Stage Disease; PREVENT‐HF – Predicting Heart Failure Risk in the Community.

Biomarkers can serve as an adjunct to clinical evaluation in large numbers of people, in a way that second‐line tests like the electrocardiogram (ECG) or transthoracic echocardiogram (TTE) cannot feasibly support at scale. Natriuretic peptides are released into the circulation directly from the myocardium exposed to increased wall stress and are widely used for the detection of HF in symptomatic people. Although brain natriuretic peptide (BNP) was identified as a key biomarker in predicting new‐onset HF in the Framingham Heart Study (FHS),^19^ and while natriuretic peptide levels increase in proportion to the severity of cardiac dysfunction,^20^ the association between BNP and HF is confounded by obesity (levels are reduced) and renal impairment (levels are increased). Elevated serum levels of BNP and NT‐proBNP are strongly associated with multiple prevalent and incident cardiovascular conditions—including, apart from HF, atrial fibrillation (AF), stroke and myocardial infarction (MI)^21^—and should therefore be interpreted within the appropriate clinical context.

Cut‐off values of 30 pg/mL for BNP and 125 pg/mL for NT‐proBNP proved to be key clinical predictors of incident HF in the community, with the population attributable risk percentage (PARP) suggesting that 18–21% of HF risk is linked to elevated natriuretic peptide levels.^22^ While BNP levels <35 pg/mL and NT‐proBNP levels <125 pg/mL can be used to ‘rule out’ HF, and a BNP concentration ≥35 pg/mL is associated with an almost 40% risk of HF,^23^ ‘ruling in’ HF remains more challenging—particularly in asymptomatic individuals. *Table* *2* lists normal ranges according to age and sex—an NT‐proBNP ≥125 pg/mL is common in females without classical cardiovascular risk factors as well as older people.^24^ The other potential barrier to this test in the recognition of early HF is cost—$42–$200 in the United States and £15–£28 in the United Kingdom, and €24.53–€40.70 are barriers to population screening.

**Table 2:** Normal ranges of natriuretic peptides, according to age and sex

Age	Female		Male	
	NTproBNP (pg/mL)	BNP (pg/mL)	NTproBNP (pg/mL)	BNP (pg/mL)
<30 years	≤196	≤55	≤104	≤29
30–39 years	≤209	≤59	≤102	≤29
40–49 years	≤233	≤65	≤137	≤38
50–59 years	≤299	≤84	≤195	≤55
60–69 years	≤399	≤112	≤333	≤93
70–79 years	≤743	≤208	≤763	≤214
≥80 years	≤2,704	≤757	≤6,792	≤1,902

A variety of approaches have been used to derive AI‐based clinical models (*Table* *3*), from classical decision trees to advanced neural networks (Residual Network [ResNet], Transformer). All studies conducted validation on independent cohorts. Most AI‐based models had a similar performance to conventional models (AUC ~ 0.8), although the BEHRT model, designed to process and analyse longitudinal electronic health record (EHR) data, achieved a high AUROC (0.93).^25^ The oRSF model,^22^ designed to handle ordinal outcomes in prognostic analysis, demonstrated the strongest discrimination in racial subgroup analyses, which is important because conventional models offer lower discrimination in population subsets defined by race. The models have not only predicted HF but also enabled the development of clinical tools (e.g., WATCH‐DM^11^). Furthermore, the models personalize the results, for example, the oRSF models incorporate race, and models based on the top 20 variables show that simplified algorithms can be equally effective. These models are getting close to real‐world implementation—developing point‐based risk scores in a step toward precision medicine.

**Table 3:** Prediction of HF with AI‐based clinical models

Study	Study population size	AI Model	Primary Outcomes	Discrimination (C‐stat, AUC)	External validation	Clinical output
Segar et al., 2019^11^	8,756 (ACCORD)	RSF	5‐year incident heart failure (hospitalization/death)	the RSF‐based model 0.77 (0.75–0.80) the WATCH‐DM risk score 0.72 (0.69–0.75)	ALLHAT the RSF‐based model 0.74 (0.72–0.76) the WATCH‐DM risk score 0.70 (0.67–0.72)	Better discrimination of the RSF‐based model than Cox models Good predictive value of the WATCH‐DM risk score
Segar et al., 2021^22^	4 cohorts: JHS: 4,141 (Black); ARIC: 7,858 (White); DHS, MESA	oRSF, Cox, Lasso, Boosted, Trees	10‐year incident heart failure (adjudicated)	0.88 (95% CI, 0.85–0.90) oRSF	yes	The oRSF Top‐20 model: superior 10‐year HF risk prediction compared to traditional HF risk and non–race‐specific ML models
Rao et al., 2021^25^	100,071 [UK CPRD: EHR]	Transformer‐ BEHRT	6‐month incident heart failure	AUROC 0.93 AUPRC 0.70	yes	Better than RETAINEX12 (in the full cohort) improvement in predictive ability: 3%–9%
Duong et al., 2021^26^	Discovery: 497,470;	XGBoost, tree‐boosting	1‐year incident heart failure (ICD codes)	AUC 0.824 (0.818–0.830)	Maine HIE AUC 0.858	Comparable to other machine learning models

ACCORD – Action to Control Cardiovascular Risk in Diabetes; ALLHAT – Antihypertensive and Lipid‐Lowering Treatment to Prevent Heart Attack Trial; ARIC – Atherosclerosis Risk in Communities Study; AUC – Area Under the Curve; AUPRC – Area Under the Precision‐Recall Curve; AUROC – Area Under the Receiver Operating Characteristic Curve; BEHRT – a deep neural sequence transduction model for EHR; CABG – Coronary Artery Bypass Grafting; Cox – Cox Proportional Hazards Model; DHS – Dallas Heart Study; HDL‐C – High‐Density Lipoprotein Cholesterol; HF – Heart Failure; HIE – Health information exchange; ICD – International Classification of Diseases; JHS – Jackson Heart Study; Lasso – Least Absolute Shrinkage and Selection Operator; MESA – Multi‐Ethnic Study of Atherosclerosis; MI – Myocardial Infarction; oRSF – Optimized Random Survival Forest; RETAINEX – Recurrent Neural Network with Attention for Interpretable Predictions; RSF – Random Survival Forest; UK CPRD – United Kingdom Clinical Practice Research Datalink; WATCH‐DM – Weight, Age, hyperTension, Creatinine, HDL‐C, Diabetes control, QRS Duration, MI, CABG; XGBoost – Extreme Gradient Boosting.

## Electrocardiographic (ECG) prediction of HF

Standard 12‐lead electrocardiography (ECG) is often done as an extension on the physical examination. ECG evidence of left ventricular hypertrophy (LVH) is associated with diastolic dysfunction,^27^ but true LVH is uncommon, and the predictive value of a negative test is poor. While a range of ECG abnormalities (e.g., arrhythmias, conduction disturbances and voltage patterns), may be seen more particularly with LV systolic dysfunction (LVD), these are not specific features. Continuous wavelet transform (CWT)‐processed ECG signals (also called energy waveform ECG [ewECG]) dissociate energy and time to explore the paradox of T wave concordance and the amplitude, duration and dispersion of the P‐wave as markers of diastolic dysfunction.^28^

In contrast, AI‐based electrocardiogram models predict HF risk with higher accuracy than can be obtained from the standard ECG (*Table* *4*). A variety of AI algorithms have been used for this purpose, including machine learning (Random forest^32^) and deep learning (convolutional neural network [CNN] or Light Gradient Boosting Machine [LightGBM]), applied to 12‐lead, single‐lead or ew‐ECGs. Fundamentally, these methods can recognize patterns in highly‐dimensional data that the human reader cannot. Models target various outcomes, including HF hospitalization, HFpEF, LV dysfunction and mortality. These studies have derived from various countries (United States, United Kingdom and Brazil), with diverse age and gender distributions, and most have undergone external validation, which supports the generalizability of their findings. Nonetheless, despite the superiority compared to the standard ECG and traditional biomarkers like NT‐proBNP (e.g., Soh et al.^37^), most of the AUCs are in the range of 0.8, although the model of Dhingra et al.^30^ achieved strong HF prediction (with a combined pooled AUC of 0.91), and Akbilgic et al.^29^ showed improved AUC when combining AI‐ECG with clinical data. A deep learning‐based algorithm predicting serum levels of NT‐proBNP from the ECG demonstrated high diagnostic and predictive value for prevalent and incident HF and other cardiovascular outcomes in the community.^21^

**Table 4:** Prediction of HF with AI‐driven ECG models

Study	Study population size	Population Demographics	AI Model Type	Primary Outcomes	Results
Akbilgic et al., 2021^29^	14,613 (ARIC)	Mean age 54 ± 5y, 45% male, United States (ARIC)	CNN,12‐lead ECG/Light gradient boosting machine (LGBM)	Incident hospitalized heart failure	CNN AUC: 0.756 (0.717–0.795) ECG‐AI model+clinical AUC: 0.818 (0.778–0.859)
Dhingra et al., 2023^30^	YNHHS: 231,285; UKB: 42,741; ELSA‐Brazil: 13,454	YNHHS (USA): 57y (IQR 42–70), 57% women; UKB: 65y (59–71), 52% women; ELSA: 51y (45–58), 55% women	AI‐ECG, 12‐lead (ECG images) + PCP‐HF	Incident HF hospitalization	AUC: 0.91 [95% CI, 0.90–0.92]) YNHHS: 0.718 (0.697–0.738); UKB: 0.769 (0.670–0.867); ELSA‐Brasil: 0.810 (0.714–0.907)
Sau et al., 2025^31^	1,163,401 ECGs 189,539 (BIDMC)	age 58 ± 19 years Boston, MA, USA	CNN, 12‐lead ECG (BRAVEHEART)	all‐cause and cardiovascular death.	Identification of sex: AI‐ECG BIDMC AUC of 0·943 [17] UK Biobank AUC 0·971 [0·969–0·972]
Potter et al. 2021^32^	398	median age 61 years, 49% women	continuous wave transforms (CWT) (ewECG)	HF risk	CWT AUC: 0.78; 95% CI: 0.70 to 0.86 CWT + ARIC AUC: 0.83; 95% CI: 0.74 to 0.92
Kaur et al. 2024^33^	326,518	Stanford Hospital, United States	CNN (12‐lead ECG)	5‐year incident HF	AUROC used, values not reported
Dhingra et al. 2024^34^	YNHHS: 194,340; UKB: 42,741; ELSA‐Br: 13,454	Similar to 2023 cohort	AI‐ECG (single‐lead)	Incident HF hospitalization	YNHHS: AUC 0.725 (95% CI 0.701–0.744); UKB: AUC 0.792 (0.696–0.889); ELSA‐Brasil: AUC 0.833 (0.720–0.946)
Gao et al. 2025^35^	238 (A), 117 (B) (validation group)	Not specified	12‐lead ECG waveform CNN‐long short‐term memory (DLM)	HFpEF risk	DLM accuracy: 78% specificity: 72% sensitivity: 72%
Choi et al. 2024^36^	1,186	Age 69 ± 15 years 57% men	CCN ECG‐GLS score	5‐year ACD and 5 HHF	Impaired LVGLS (≤ 12%) AUROC 0.82; sensitivity, 85%; specificity, 59% LVEF < 40% AUROC 0.85
Soh et al. 2024^37^	275 community‐based patients at risk of HF	Age 70–72, 42% women	12‐lead ewECG CWT	Primary: SBHF secondary: DD; reduced GLS and LVH	SBHF ewECG AUC of 0.81 (26) NT‐proBNP AUC 0.56 [0.44–0.68], *P* < 0.001 ARIC HF AUC 0.67 [0.56–0.79], *P* = 0.002 DD AUC 0.74 [0.73–0.74] reduced GLS AUC 0.76, 95% CI 0.73–0.74 LVH AUC 0.90, 95% CI 0.88–0.89
Neyazi 2024^21^	HCHS: 8,253 patients	Age 45–74, 50,9% women	1D ResNet CNN	Prediction of NT‐proBNP serum levels from ECG; risk assessment for heart failure and other cardiovascular diseases	HCHS: Correlation R = 0.566; AUROC for prevalent HF = 0.795; predictive performance comparable to measured NT‐proBNP SHIP‐START‐0: Correlation R = 0.642; AUROC for prevalent HF = 0.816; strong generalizability SHIP‐START‐1 (5 y follow‐up): AUROC for incident HF = 0.669; predictive value comparable to measured NT‐proBNP SHIP‐START‐2 (10,8 y follow‐up): AUROC for incident HF = 0.709; stroke = 0.800; AF = 0.854; MI = 0.798; CV death = 0.895 SHIP‐START‐3 (15,7 y follow‐up): AUROC for incident HF = 0.708; stroke = 0.806; AF = 0.881; MI = 0.783; CV death = 0.881 SHIP‐TREND‐0: Correlation R = 0.655; AUROC for prevalent HF = 0.783; strong generalizability; SHIP‐TREND‐1 (7,3 y follow up): AUROC for incident HF = 0.689; stroke = 0.703; AF = 0.858; MI = 0.788; CV death = 0.821

ACD – Advanced Cardiac Diagnostics; AI – Artificial Intelligence; ARIC – Atherosclerosis Risk in Communities Study; AUROC – Area Under the Receiver Operating Characteristic Curve; AUC – Area Under the Curve; BIDMC – Beth Israel Deaconess Medical Center; BRAVEHEART – Biomarker Risk Assessment in Vulnerable Elderly for Heart Health Evaluation and Risk Tracking; CNN – Convolutional Neural Network; CWT – Continuous Wavelet Transform; DD – Diastolic Dysfunction; DLM – Deep Learning Model; ECG – Electrocardiogram; ELSA‐Brazil – Brazilian Longitudinal Study of Adult Health; HCHS – Hamburg City Health Study; HHF – Hospitalization for Heart Failure; LGBM – Light Gradient Boosting Machine; LVGLS – Left Ventricular Global Longitudinal Strain; LVEF – Left Ventricular Ejection Fraction; LVH – Left Ventricular Hypertrophy; PCP‐HF – Pooled Cohort Equations to Prevent Heart Failure; ResNet – Residual Neural Network; SBHF – Subclinical Biventricular Heart Failure; SHIP – Study of Health in Pomerania; UKB – UK Biobank; YNHHS – Yale New Haven Health System.

In general, the performance of AI‐powered ECG analysis in recognizing asymptomatic LV dysfunction is less than that for the prediction of HF. Nonetheless, the findings suggest that this approach could serve as a scalable tool for early HF risk detection, potentially enabling widespread community‐based screening programmes.

## Echocardiographic recognition of HF

Echocardiographic imaging is the cornerstone of HF detection, phenotyping and management. From the standpoint of HF screening, there are three challenges. Image acquisition requires a highly‐trained sonographer or cardiologist. Accurate measurements are required for recognition of SBHF, including LV dimensions and wall thicknesses, mass, volumes, EF from two‐ (2D) or three‐dimensional (3D) techniques, LV and LA strain, left atrial dimension and volume, mitral inflow (E/A ratio, DT and IVRT), annular velocities and tricuspid regurgitant velocity. Finally, patients at risk of HF are typically elderly and multimorbid, necessitating comprehensive echo evaluation, which requires expertise and time for interpretation. All of these steps have been facilitated by AI.

Emerging data support the role of AI guidance in enhancing echocardiographic image acquisition.^38^ In studies involving novice users (nurses and medical residents with no prior imaging experience), AI systems facilitated the capture of diagnostic‐quality images for key cardiac parameters, with success rates ranging from 81% to 94% depending on the specific view. This compares favourably to the 88% to 100% success achieved by experienced sonographers. AI‐driven automatic measurements of LV volumes and ejection fraction may yield substantial time savings, including a 77% reduction in scan time (median saving of 5.3 min) and a decrease in scan duration from 85 to 57 s.^39^ In educational settings, trainees achieved improved diagnostic quiz scores (85% with AI vs. 50% without) and increased confidence, approaching expert performance levels.^39^

Deep learning models have been used to identify HF risk using echocardiography. These have mainly been convolutional neural networks (including 3D and spatio‐temporal variants), along with LSTM and radiomics approaches (*Table* *5*). The reported sensitivities range from 85% to 96% and specificity from 85% to 91%, with area under the curve values between 0.85 and 1.0. Several studies have compared AI‐generated outputs with traditional clinical scoring systems (e.g., H2FPEF and HFA‐PEFF) and expert interpretations, demonstrating that AI algorithms can effectively reclassify indeterminate cases and, in certain instances, outperform conventional risk assessment tools.^42^, ^47^ These findings indicate that AI‐driven echocardiography can deliver rapid, automated and accurate assessments for HF prediction when applied under varied clinical settings.

**Table 5:** Prediction of HF with AI‐driven echocardiography models

Study	Study population size	AI model type	Primary outcomes	Performance (AUC)	Clinical Impact
Chiou et al., 2021^40^	1041 patients with HFpEF 1263 asymptomatic individuals	CNN on 4‐chamber echocardiography, intrabeat dynamics	HFpEF detection	Internal: 0.96/0.85; External: 0.79/0.89 Overall: AUC 0.95 Accuracy 0.91, sensitivity 0.96, specificity 0.85	Accurate prescreening method, time‐saving, facilitates diagnosis, quantitative metrics
Lau et al., 2023^41^	27 135 (64 028 echos)	3D CNN	Automated echo quantification, prediction of CV outcomes	AUC > 0.97 for PLAX, A4C and A2C views vs human expert annotation Quantified standard measures (R^2^ range 0.53–0.91 vs study report)	Enables large‐scale disease prediction (HF, AF, MI and death)
Ackerman et al. 2023^42^	6756 (2971 cases)	3D CNN on apical 4‐chamber video	HFpEF classification	Training AUROC 0.97 Validation AUROC 0.95 HFA‐PEFF score sensitivity 95% specificity 97% AI HFpEF model sensitivity 87% specificity 71%	High sensitivity/specificity; supports HFA‐PEFF scoring
Liastuti et al., 2022^43^	138	LSTM (LIFES) on echocardiography video compared to expert diagnosis	Rapid HF diagnosis	AUC A4C 0.89, A2C 0.93, PLAX 0.84 Sensitivity/specificity for discriminating between HF and normal patients: 2CH 96%/87%, 4CH 96%/80%, PLAX 95%/77%	Fast, feasible, accurate and diagnosis
Liu et al., 2023^44^	8976 images, 10 085 videos	R2plus1d‐Pan (spatio‐temporal CNN)	HFrEF, (<40%) detection	AUC training 0.95 AUC validation 1.0	Matches/exceeds expert performance
Pandey et al., 2021^45^	1242 + TOPCAT validation	DeepNN	HFpEF phenogrouping, LV filling pressure, outcomes	Training AUROC 0.988, Internal validation AUROC 0.997, After excluding other than e′ LV diastolic function parameters AUROC 0.945 to 0.997	Identifies high‐risk phenotypes and guides therapy
Huang et al., 2024^46^	100 symptomatic,	Us2.ai (commercial deep learning platform)	Detection of reduced LVEF (<50%) by novices	AUC 0.88 (95% CI 0.80–0.96) Sensitivity 85%, specificity 91%, positive predictive 79% and negative predictive 94%	Task‐shifting and high negative predictive value for screening

3D CNN, three‐dimensional convolutional neural network; A2C, apical two‐chamber view; A4C, apical four‐chamber view; AF, atrial fibrillation; AUC, area under the curve; AUROC, area under the receiver operating characteristic curve; CNN, convolutional neural network; CV, cardiovascular; DeepNN, deep neural network; HF, heart failure; HFA‐PEFF scoring, Heart Failure Association, Preserved Ejection Fraction Diagnostic Scoring System; HFpEF, heart failure with preserved ejection fraction; HFrEF, heart failure with reduced ejection fraction; LSTM (LIFES), long short‐term memory (learning‐based inference framework for echocardiographic sequences); LV, left ventricle; MI, myocardial infarction; PLAX, parasternal long axis view; R2plus1d‐Pan, Residual 2 + 1D Spatio‐Temporal Convolutional Neural Network (Pan architecture); TOPCAT, Treatment of Preserved Cardiac Function Heart Failure with an Aldosterone Antagonist Trial; Us2.ai, automated echocardiography analysis platform.

*Table* *5* presents a structured analysis of the echocardiography‐based AI studies, focusing on model performance, clinical relevance and innovation. The predominant methodology across these studies involved convolutional neural networks (CNNs), with a growing incorporation of 3D and spatio‐temporal architectures. Models such as LSTM and DeepNN show considerable potential for dynamic video analysis and phenotyping. In summary, the highest model performance (AUC) was achieved by Pandey et al.^45^ (up to 0.997), Liu et al.^44^ (validation AUC 1.00), Lau et al.^41^ (>0.97 across views). The most robust external validation was reported in studies by Chiou^40^ and Akerman,^42^ with AUC values in validation cohorts ranging from 0.89 to 0.95. Furthermore, Huang et al.^46^ demonstrated that AI can enable non‐experts to achieve high diagnostic accuracy, while Pandey et al.^45^ highlighted its potential in stratifying patients with HFpEF for targeted treatment. The feasibility of AI for large‐scale deployment in echocardiography labs was notably demonstrated in studies by Lau et al.^41^ and Liu et al.^44^ Platforms such as Us2.ai and LIFES further underscore the commercial promise of AI and its integration into handheld devices.

## Implications and Conclusions

Recent years have witnessed development and expansion in application of AI in medicine, particularly in image acquisition and interpretation, and disease prediction. This paper reviewed the feasibility of implementing AI‐based clinical, ECG and echocardiographic approaches for detecting individuals at risk of HF. Clinical AI models have demonstrated effectiveness in predicting incident heart failure (HF) across diverse populations and time horizons. While all analysed models achieved good discrimination metrics, the highest predictive performance was observed with the BEHRT model (AUC 0.93).^25^ Its transformer architecture, which analyses sequential data from electronic health records (EHR), makes it particularly amenable to screening applications. The oRSF model^22^ achieved an impressive C‐index of 0.88 for 10‐year HF prediction, highlighting its potential for long‐term risk monitoring. These models appear robust, having been externally validated and tested on large, heterogeneous populations.^26^ Moreover, their architectural flexibility provides a foundation for incorporating additional signals, such as ECG or echocardiography, thereby expanding their clinical utility. The inclusion of different ethnic groups^22^ addresses the need for equity in healthcare. Some models have been transformed into conventional tools for clinical use, such as WATCH‐DM^11^ (9 cLinical variables) and oRSF^22^ (20 variables). AI‐powered ECG analysis shows encouraging potential to enhance the diagnostic value of both clinical and conventional ECG assessments.

**Figure 2:**
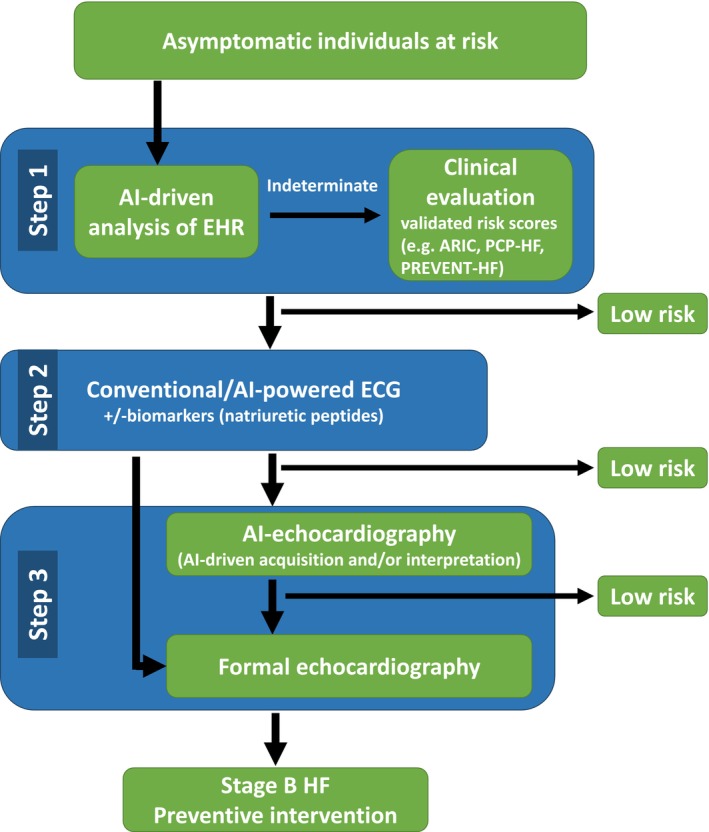
Proposed structured approach to prediction HF risk in asymptomatic individuals.

*Figure* *2* outlines a structured approach to predicting HF risk in asymptomatic individuals. The initial step involves AI‐driven analysis of EHR. In cases where this assessment yields indeterminate results, a comprehensive clinical evaluation using validated risk scores should be undertaken to enhance risk stratification. Individuals identified as having greater than low HF risk in this stage should proceed to conventional or AI‐powered ECG, assisted by biomarkers (natriuretic peptides). Following the exclusion of low‐risk individuals in this second step, the remaining cohort advances to the final stage: echocardiographic assessment. Depending on local resource availability, two pathways may be considered—either direct referral for formal echocardiography or an additional filtering phase involving AI‐driven image acquisition and/or interpretation. This intermediate step allows for further identification of low‐risk individuals, thereby reducing the number of subjects requiring formal echocardiographic evaluation. The proposed three‐step framework aims to optimize early identification of individuals at elevated risk for HF, supporting timely and targeted preventive interventions.

Existing data confirm that artificial intelligence holds real potential to transform care for patients at risk of HF. Ensuring model interpretability, integration with EHR systems, validation across diverse clinical settings, and, crucially, the education of medical personnel in the use of AI technologies will be key to further development.

## Funding

This work was supported in part by the National Health and Medical Research Council, Canberra, Australia (Investigator grant 2008129).

## Appendix A

**Table A1:** Supervised approaches to machine learning.

Type of ML model	Description
Regression analysis	Statistical method to model the relationship between a dependent (the outcome) with one or more independent variables (predictors).
Support vector machines	Algorithm that separates data points into different classes by finding an optimal hyperplane with the maximum margin between the classes. Application to: classification, regression, outlier detection
Random forests (RF)	Algorithm that combines multiple decision trees to make predictions or classifications by aggregating the outputs of individual trees to improve prediction accuracy and control overfitting. Application to: classification, regression
Artificial neural networks (ANN)	Collection of connected units called artificial neurons, which loosely model the neurons in a biological brain. Each neuron receives input, applies a mathematical transformation and passes the result to the next layer. Application to: disease prediction and diagnosis, drug discovery and development, personalized treatment recommendations.
Convolutional neural networks (CNN)	Deep learning architecture specifically designed for processing grid‐like data, such as images, by using convolutional layers to automatically learn hierarchical patterns and features. Application to: image analysis
Deep learning (DL)	DL is defined as a class of artificial neural network algorithms, in which more internal layers are used than in traditional neural network approaches (‘deep’ merely describes a multilayered separation approach). Often described as convolutional neural networks. Application to: image analysis, diagnosis and prediction

**Table A2:** Unsupervised approaches to machine learning.

Type of ML model	Description
Principal component analysis (PCA)	Used for the dimensionality reduction in ML; converts the observations of correlated features into linearly uncorrelated features with orthogonal transformation. Application to: reduce complexity, improve visualization, remove noise, avoid multicollinearity.
Partitioning algorithms	An iterative algorithm which groups the unlabelled dataset into different clusters. Here K defines the number of pre‐defined clusters that need to be created in the process. Advantages: simple and easy to implement, interpret, fast, and efficient. Has remained relatively underutilized in cardiology despite its simplicity In implementation and interpretation. Disadvantages: uniform cluster size, may work poorly with different densities of clusters, difficult to find k, sensitive to outliers.
Hierarchical clustering	Algorithm that groups data based on similarity with another cluster by aggregating or dividing them as it moves in hierarchy—tree‐like structure called a dendrogram. Advantages: easy comprehension and illustration using dendrograms, insensitive to outliers. Disadvantages: arbitrary metric and linkage criteria, does not work with missing data, may lead to misinterpretation of the dendrogram, difficulty finding optima solution.
Model‐based clustering	Algorithm makes a general assumption that the data in each cluster is generated from probabilistic (primarily Gaussian) model. Advantages: distributional description for components, possible to assess clusters within‐clusters, can make inference about the number of clusters. Disadvantages: computationally intensive, can be slow to converge.
Grid‐based algorithms	Algorithm arranges data into a grid structure and identifies clusters by analysing density variations within the grid. Advantages: can work on large multidimensional space, reduction in computational complexity, data space partitioned to finite cells to form grid structure. Disadvantages: difficult to manage irregular data distributions; limited by predefined cell sizes, borders, and density threshold; difficult to cluster highdimensional data.
Density‐based spatial clustering of applications with noise (DBSCAN)	Algorithm identifies clusters of any shape and can handle noise and outliers. Advantages: does not require number of clusters, can find clusters of arbitrary shapes, robust to outliers. Disadvantages: not deterministic, quality depends on the distance measure, cannot manage large differences in densities.
